# Multimorbidity and equity in health

**DOI:** 10.1186/1475-9276-12-59

**Published:** 2013-08-20

**Authors:** Efrat Shadmi

**Affiliations:** 1 Faculty of Social Welfare and Health Sciences, University of Haifa, Mount Carmel, Haifa 31905, Israel

## 

Two major challenges are at play in the design of current and future health care delivery: the growing diversity of patient populations and the increasing burden of multiple long-term conditions. The growing prevalence of multiple co-occurring conditions signifies a greater burden for patients and a resultant increasing need for health care resources [1,2]. Yet, health care systems are ill suited for the complex, frequently interacting needs of patients with multiple chronic conditions [3]. Guidelines and incentives are aligned in a way that acknowledge high quality treatment of single conditions [4,5] without recognizing the impact of multiple conditions on the commissioning of care and on patients’ self management capabilities [6-9]. For individuals from diverse socioeconomic and cultural backgrounds, these challenges are even greater, as multiple co-occurring life and social circumstances interact with their health and health related needs.

## Definitions

Assessing the effects of multimorbidity (MM), the co-occurrence of several conditions, is contingent on the way it is measured [10,11]. Current definitions usually apply an additive approach, summing chronic diseases out of predetermined lists [11]. A wide range of variation within single chronic conditions can be explained by diversity in the co-occurrence of related conditions, sequel of the condition of interest (e.g., complications), the stage of the disease, and presence of other “non-related” conditions [12,13].

To effectively reflect the burden of morbidity, beyond a simple count or “list” of the common chronic conditions that are present in individuals or populations, valid methodologies that take into account combinations and interactions between conditions are available [14]. These classification systems categorize patients’ morbidity according to all diagnoses registered at medical encounters during a defined period of time (usually a year). Relying on clinical and epidemiological considerations, diagnoses are classified into morbidity groups, spanning from minor acute to major chronic illnesses, with various degrees of severity. Previous studies have shown that encompassing morbidity as part of a complex morbidity measurement system is highly reliable and valid, in various countries and population groups [15].

Nonetheless, even the existing comprehensive measures of morbidity fail to capture the full complexity of states that are related to a persons’ health, as they are based only on clinical diagnoses. An emerging construct is that of *patient complexity* which acknowledges that morbidity burden is influenced not only by health-related characteristics, but also by socioeconomic, cultural, environmental, and behavioral attributes [10].

A broader definition of MM is needed also because diseases are more likely to occur and to be more severe in socially disadvantaged populations [16,17]. Inequity in health, defined as “differences in health which are not only unnecessary and avoidable but, in addition, are considered unfair and unjust” [18], is at the focus of societies and health systems worldwide. Despite impressive improvements in the health status of populations, there is increasing evidence of widening health gaps that span the socioeconomic spectrum and range of minority ethnic groups [19-21].

## The interrelatedness of equity and multimorbidity

Individuals from socially diverse populations face unique health and health related problems which add to the complexity of multimorbid care, with care systems that are, largely, inadequate in terms of financial, organizational and linguistic accessibility and cultural competency. While current research and practice provide some direction for the care of multimorbid or diverse populations, health and social policy requires new multidisciplinary frameworks for understanding the interplay between the key dimensions affecting health and health care of multicultural multimorbid older adults. Only recently has the literature on MM begun to address the socio-cultural aspects of patient care [22]. Still, the role of health care organizations in reducing health inequity is far from being realized [23,24].

The pathways between social-economic disadvantage and poor health have been extensively studied [25]. Patients from minority groups face challenges related to low language proficiency and poor health literacy [26]. Minority patients often receive care from culturally and ethnically discordant providers not familiar with their cultural norms and beliefs, which creates challenging care encounters and difficulties in supporting self management [27]. Some social groups, for example, new immigrants, experience low social cohesion and social capital [28]. With few social ties, stress may accumulate, resources for support may be difficult to access and health may be negatively affected.

Similarly, low socioeconomic populations face significant financial barriers to accessing care, and are even more significantly affected when they have multiple conditions to manage. When accessibility is limited, out-of-pocket expenditures [29], coordination of various treatment recommendations from multiple providers [30], ensuring that appointments are not missed and effectively navigating the health care system can be extremely challenging [31,32].

The disciplinary disconnect in the realms of MM and equity may partly explain the dearth of research on socio-cultural aspect affecting care of multimorbid patients. The scarce literature that does exist, however, depicts the complexity involved in the care of multicultural multimorbid adults. For example, qualitative interviews with physicians and nurses on managing the care of multimorbid patients in deprived areas describe the “endless struggle” of trying to manage illness in the midst of chaotic lives with limited resources and multiple, competing needs [33]. Additionally, areas of parallel investigation point to the need for an all-inclusive view of the challenges posed by MM in ethnically diverse populations. Self management is challenging for persons with MM, as adhering to complex care management regimes for multiple co-occurring conditions may be overwhelming [34]. Research on self management shows that under-served and minority populations face multiple challenges (such as low language proficiency or provider-patient discordance) [35]. Yet, research to date, does not provide a comprehensive guide for understanding the overall interrelated influences of multiple health conditions and multiple cultural aspects.

## A special series on equity and multimorbidity

This special series aims to contribute to the growing body of knowledge on MM and equity in diverse populations worldwide. The inaugural issue, which will be followed by ongoing publications, presents 11 papers from various (World Health Organization) regions - Eastern Mediterranean countries [36] America [37,38], Africa, [39,40], Europe [41-44], and the Western Pacific [45].

In a review of MM and equity in Eastern Mediterranean countries, Boutayeb and colleagues [36] summarize findings from 26 studies, showing that female gender, low income, low level of education, and unemployment are factors associated with MM. Given the rising prevalence of non-communicable diseases (NCDs) in the region [46], there is also increased awareness that NCDs do not occur in isolation, and studies on the prevalence and risks associated with multiple NCDs are emerging. The main outstanding areas of contribution that authors point to include: expanding geographic scope (particularly, studies from North Africa), types of conditions included in MM measurements and types of socioeconomic and ethnic/cultural risk factors examined. Additionally, it should be noted that the study of MM in low and middle income countries in general, and specifically in Eastern Mediterranean countries, is still mainly focused on co-morbidity, i.e., the co-occurrence of disease or conditions in the context of an index condition of interest [10], rather than on MM. To better understand patterns of MM, their causes and consequences, there is the need to expand this conceptualization toward acknowledging that the overall burden of co-occurring and interacting conditions is not necessarily associated with a single disease in various population groups.

The two studies from South Africa [39,40] further emphasize the relative robustness of the relationship between deprivation and MM. Each used a different survey with distinctive measures of MM and deprivation, and both concluded that income is inversely associated with MM. It is important to note, however, that comprehensive measurements (i.e. those that combine reports on both illness and disability) [40] may provide a more informative depiction of the phenomenon, thereby guiding further research and policy.

In a study from Taiwan, Kuo and colleagues [45] show that lower income individuals with MM incur higher total costs. Several possible explanations for this relationship are discussed by the authors. Future studies should examine this type of relationship with a more comprehensive MM measure that accounts for all types of conditions and their interactions encompassing more than the additive effect of specific conditions [47]. A more comprehensive MM measure might be able to better capture the multitude of health and health related needs which are more prevalent among disadvantaged populations, and which are potentially driving up total expenditures in this multimorbid population group.

That MM encompasses more than just chronic conditions is exemplified in the study by Reis-Santos and colleagues [37], who characterized MM in subjects with tuberculosis (TB) registered in the National Notification System in Brazil. This study, however, found that MM in persons with TB was not associated with deprivation indices such as education level or prior Institutionalization (e.g., imprisonment).

Three studies in this issue address another area of needed research: MM and equity in childhood and adolescence [38,41,42]. Cornish and colleagues [42] have demonstrated a link between levels of maternal education and MM among children, but not in adolescents. This study used several types of MM measures, including the Adjusted Clinical Groups® classification of morbidity burden, taking into account all types of conditions (chronic and acute) and their interactions [48]. The inconsistency of results across the various types of measures suggests that additional work is needed in development of both the conceptual framework as well as methodology of studies assessing MM, especially in children and youth [49]. Chao and colleagues [41] present a complimentary approach to the study of MM, in which they conducted a survey on the prevalence of multiple mental health, and behavioral difficulties (i.e. the consumption of alcohol, tobacco, cannabis, and hard drugs, obesity, depressive symptoms, suicide attempts, involvement in violence, and low school performance) in middle schools in north-eastern France. Although the representativeness of this sample should be further established, this study contributes new insights about the co-occurrence of a wide range of physical and mental health conditions, coupled with socio-economic deprivation in adolescents.

The third paper that studies children uniquely examines MM and the association with residential movement patterns and changes in neighborhood income of children with mild to severe chronic diseases compared to healthy children [38]. This study shows that young children with chronic conditions, particularly those born in low income neighborhoods, are more likely to move residence than other healthy young children. The reasons for--as well as outcomes of--such mobilization requires further research.

Another important contribution to the study of MM and equity is presented in a paper by Lawson and colleagues [43], which studied the association between MM and Health Related Quality of Life (HRQoL) and its variation by socioeconomic deprivation. This study found that MM has a substantial negative impact on HRQoL which is most severe in areas of deprivation, especially among younger adults. Given that young adults are prone to the negative effects of deprivation on MM, this constitutes an essential area for further investigation, especially as most current research on MM is still focused on older adults [49-52].

Another distinct area of inquiry in the field of MM is the study of incidence rather than prevalence of co-occurring conditions. While most research focuses on describing the prevalence of MM, several studies also point to the relationship between deprivation and onset of MM [53,54]. Demirchyan and colleagues add to this limited body of knowledge in a study on the determinants of incident MM in a cohort from the 1988 Armenian earthquake survivors [44]. This prospective study was able to show that the relationship between age and MM was largely mediated by the number of stressful life events, suggesting that experiencing stressful events during the lifespan might be a more important factor for incident MM than the lifespan itself. This study also suggested that BMI is an independent long-term predictor of incident MM, with higher BMI values contributing to MM two decades later--a finding, that as the authors justly state, deserves attention and further study.

Finally, this inaugural issue of the thematic series on equity and MM also includes a commentary [55] on equity in the selection of MM patients for inclusion in targeted care-management interventions. Multimorbid patient selection is complicated due to the lack of clear criteria - unlike disease management programs for which patients with a specific condition are identified. This ambiguity can potentially result in inequitable selection, as biases in selection may differentially affect patients from disadvantaged population groups. Acknowledging these biases is an important first step for preventing further widening of gaps in the care of multimorbid individuals.

## The future of research on equity and multimorbidity

To date, most studies on equity and MM are descriptive or correlational, addressing specific risk factors, often using cross-sectional designs. Further research is needed to address the full range of populations, combining economic, social, cultural and ethnic characteristics, to enable a better understanding of the types of populations that are affected by MM. Specific populations that would benefit from additional investigation are younger, working age adults, children and adolescents. As most of the research on MM is focused on older adults, investigating the occurrence of MM in deprived populations, with generally shorter life expectancy, a multitude of challenges to maintaining healthy life styles, and difficulties in accessing high quality health care, requires a shift towards younger age groups.

Similarly, the measurement of MM needs to be broadened. Defining MM as the sum of chronic conditions is a limited characterization that may fail to capture the burden associated with MM, encompassing chronic as well as acute conditions and risk factors. A narrow, additive classification of MM may limit the identification of the magnitude of differences in MM between varying populations, and may underestimate the effects of inequitable care in MM populations.

Future research should also address the outcomes of MM in deprived populations. Initial findings suggest a differential effect of MM on outcomes by population characteristics. Why disadvantaged individuals with the same levels of MM as non-disadvantaged individuals achieve poorer outcomes is an important question to address in the quest for preventing the widening gaps and inequity in health.

Finally, targeted policies and interventions for preventing and treating MM in persons from diverse socio-economic and cultural backgrounds are urgently needed. As primary care provides a whole-person, comprehensive approach to care, it is the setting most appropriate to focus care provision for persons with diverse multiple health and health related needs [56]. Ultimately, a broad body of knowledge will aid in directing policy and practice towards a better understanding of the root causes of inequity in MM and the refinement of methods for its eradication.
